# Knockdown MTDH Inhibits Glioma Proliferation and Migration and Promotes Apoptosis by Downregulating MYBL2

**DOI:** 10.1155/2022/1706787

**Published:** 2022-09-12

**Authors:** Junqi Fu, Jun Peng, Guolong Tu

**Affiliations:** Department of Neurosurgery, Haikou People's Hospital, Haikou, Hainan Province 570208, China

## Abstract

Glioma is a malignant tumor that often occurs in the adult central nervous system. Metadherin/astrocyte-elevated gene-1 (MTDH) is involved in the development of cancer, but its relationship with glioma remains unclear. This study is aimed at clarifying the role of MTDH in glioma. GEPIA was employed to find the difference of the expression level of MTDH and MYB protooncogene-like 2 (MYBL2) in glioma tissues and normal tissues, and real-time quantitative reverse transcription PCR (qRT-PCR) and western blot (WB) were applied to verify the differential gene expression of MTDH and MYBL2 cells. After knocking down of MTDH, the expressions of forkhead box M1 (FoxM1), MTDH, and MYBL2 were detected by WB cells. Cell counting kit 8 (CCK-8) was used to detect cell proliferation, and flow cytometry was applied to measure cell apoptosis. The transwell assay was utilized to investigate the ability of cell migration and invasion. The results showed that MTDH and MYBL2 were overexpressed in glioma cells compared with normal cells. The knockdown of MTDH would inhibit the expression of MYBL2 through decreasing the expression of FoxM1 and further reduce glioma cell proliferation and cell migration and invasion. The present study showed that knockdown of MTDH inhibits glioma proliferation and migration and promotes apoptosis by downregulating MYBL2, which suggests that MTDH is a potential gene in clinical treatment of glioma.

## 1. Introduction

Glioma is a common malignant primary brain tumors in the adult central nervous system [1]. Gliomas are not only common but are also aggressive among primary brain tumors and have a poor prognosis after treatment. However, due to the particularity of the structure of the central nervous system of the brain, there are many challenges to drug development. Despite recent advances in diagnostic modalities and treatment strategies, the mortality of gliomas patients remains high [2]. Epidemiology statistics analysis showed that the survival rate of glioma patients in five years is relatively low among cancers [3, 4]. The main obstacle to the current treatment is the immunosuppressive tumor environment (TME), and the second is drug resistance [5]. Because it occurs primarily through genetic modification, gene therapy, which currently alters the genetic makeup of target cells, will be incomparable among current therapeutic strategies. Alternative treatments for glioma are limited because the underlying pathophysiological mechanisms of disease are unknown. Therefore, understanding of the specific mechanisms of glioma initiation and progression will provide schemes for accurate diagnosis, early intervention, and useful treatment.

Metadherin/astrocyte-elevated gene-1 (MTDH) (also known as astrocyte-elevated gene-1/LYRIC) is an abnormally activated oncogene found in a variety of cancers. It is a multifunctional protein, and the gene encoding MTDH is located on chromosome 8q22. Its main function is to promote tumor growth and migration, and it is also related to drug resistance. In addition, MTDH can also regulate oxidative stress, immune aging, and other functions [6]. MTDH is not overexpressed in normal people, but when people develop cancerous lesions or even advanced stage, the expression of MTDH gene may be highly activated, which directly affects clinical prognosis [7]. According to *in vivo* and *in vitro* experimental studies, overexpression or knockdown of MTDH can affect a series of cell activities such as gene expression and cell proliferation. Moreover, the invasive and metastatic capabilities of tumor are also altered by MTDH [8]. Recent studies have shown that high levels of MTDH expression are observed in patients with glioblastoma, and these patients have a poor prognosis [9]. However, it is still unclear how MTDH regulates glioma and its target gene [10–12].

The MYB protooncogene-like 2 (MYBL2) gene is a member of the myeloblastosis transcription factor family [13]. MYBL2 plays a critical role in cell proliferation and regulation of cell differentiation. Besides, it is also crucial in directing cell cycle progression [14]. Meanwhile, it was overexpressed in cancers such as myeloid leukemia [15], hepatocellular carcinoma [16], and breast cancer [17]. Recent studies have demonstrated that low expression of MYBL2 induces cell cycle arrest, apoptosis, and epithelial–mesenchymal transition (EMT) in glioma cells, and it is positively regulated by FoxM1, a member of the forkhead box transcription factor family [18]. MTDH enhances the stability and transcriptional activity of FoxM1 [5]. Whether MTDH positively regulates MYBL2 in glioma cells needs further study. This study is aimed at investigating the role of MTDH in glioma.

## 2. Methods

### 2.1. Cells and Regents

Human brain astroglial normal cell HEB and glioma cell lines U251, U87, T98G, and LN-229 were obtained from Zhejiang University (Hangzhou, China). Cells were cultured in DMEM (Solarbio, Beijing, China) supplemented with 10% FBS (Gibco, CA, USA) and 1% of penicillin-streptomycin (Arizona, USA). Cells were cultured at 37°C in a cell incubator containing 5% CO_2_.

### 2.2. Expression Analysis by Gene Expression Profiling Interactive Analysis (GEPIA)

GEPIA (http://gepia.cancer-pku.cn/index.html) is an online database. It was applied to compare and study the expression level of MTDH and MYBL2 in glioma and normal tissues. Boxplot with disease status (tumor or normal) was used as a variable to calculate the differential expression of MTDH and MYBL2.

### 2.3. Lentiviral Transfection

MTDH lentiviral-based gene silencing particle was purchased from Beyotime (Nanjing, China) and was cloned into GV115 plasmids. The sequences of MTDH silencing RNAs were as follows: siMTDH-1, 5′AGGAATAAAGGATTCTGAT3′; siMTDH-2, 5′-AAGTCAAATACCAAGCAAA-3′; and siMTDH-3, 5′-AACTTACAACCGCATCATT-3′, as well as a negative control siRNA (siNC), 5′-TTCTCCGAACGTGTCACGT-3′ [19]. The grouping scheme was as follows: (1) the control group, (2) the NC group (noncoding sequence control), (3) the pc-MTDH group (overexpressing MTDH), (4) the siNC group (empty transfection), and (5) the siMTDH (MTDH knockdown) group. Subsequently, with the help of packaging plasmid (10 *μ*g) and the transfer plasmid (15 *μ*g), the GV115 plasmid (20 *μ*g) was cotransfected into 293T cells using Lipofectamine® 2000 (Solarbio, Beijing, China). Following transfection with the lentiviruses for 48 h (MOI = 10), the transduced cells were selected with 5 *μ*g/ml puromycin (Solarbio, Beijing, China). The grouping scheme was as follows: (1) the HEB group, (2) the U251 group, (3) the U87 group, (4) the T98G group, and (5) the LN-229 group. After knockdown of gene, the grouping scheme was as follows: (1) the siNC group, (2) the siMTDH group, (3) the siMTDH+NC group, and (4) the siMTDH+MYBL2 group.

### 2.4. Real-Time Quantitative Reverse Transcription PCR (qRT-PCR)

A SteadyPure Plant RNA Extraction Kit (Hunan, China) was used to lysate cells to extract total RNA. Then, cDNA was synthesized by a universal RT-PCR kit (Solarbio, Beijing, China). SYBR Green Master Mix (Bio-Rad, USA) was employed for the qRT-PCR, and *β*-actin was used as an internal control. The grouping scheme was as follows: (1) siNC+pc-control, (2) siMTDH+pc-control, and (3) siMTDH+pc-FoxM1. All primers used are listed in Table 1.

### 2.5. Western Blot

Protein was lysed from cells by using RIPA buffer (Cat no. 89901; Thermo Fisher, MA, USA) followed by centrifugation at 16,000 × *g* and 4°C for 15 min. To quantify the protein concentration, a bicinchoninic acid (BCA) protein assay (Cat no. 23225; Thermo Fisher, MA, USA) was performed. Denaturation of total protein was processed in a metal bath at 95°C for 5 min, and then, 30 *μ*g of total proteins was separated by SDS-PAGE (7.5%). After that, proteins were transferred to PVDF membranes. 5% skimmed milk was dissolved in TBST to block the PVDF membrane for 2 h at room temperature. Then, the membranes were washed and incubated with the primary antibodies overnight.

The primary antibodies included anti-MTDH (1 : 1500) (Cat no. 13860-1-AP; Proteintech, Wuhan, Hubei, China), MYBL2 (1 : 1500) (Cat no. 18896-1-AP; Proteintech, Wuhan, Hubei, China), FoxM1 (1 : 1500) (Cat no. 13147-1-AP; Proteintech, Wuhan, Hubei, China), and *β*-actin (1 : 2000) (Cat no. CL488-66009; Proteintech, Wuhan, Hubei, China) antibodies. Then, the membranes were incubated with the horseradish peroxidase- (HRP-) conjugated secondary antibody (1 : 1000) (Cat. no. 7074; Cell Signaling Technology, Inc.) for 2 h at 37°C. To measure the chemiluminescence signals, the EasySee Western Blot Kit (Beijing TransGen Biotech, Beijing, China) was utilized. ImageJ version 1.53 software was used to quantify the proteins.

### 2.6. CCK-8

CCK-8 assay (Solarbio, Beijing, China) was maneuvered to measure cell viability of cells with indicated transfections. Cell proliferation rates were measured after 24 hours. The absorbance value could be immediately read at 450 nm by a microplate reader (SpectraMax i3X, Molecular Devices, CA). At each concentration, each group was repeated for three times, followed by data analysis.

### 2.7. Flow Cytometry

To evaluate the apoptosis rates of cells, a BD Accuri™ flow cytometer (BD Biosciences, Franklin Lakes, NJ, USA) was employed to flow cytometry. An Annexin V-fluorescein isothiocyanate (FITC)/propidium iodide (PI) apoptosis detection kit (Solarbio, Beijing, China) was applied for detecting the proportions of cell cycle and cell apoptosis, and then, results were analyzed according to manufacturer's protocols. The ratio of cell apoptosis was computed and calculated using FACS scan software (BD, San Jose, USA).

### 2.8. Colony Formation

When cells grown to logarithmic growth period, cells were subcultured and evenly planted in a plate and cultured in an incubator at 37°C. Colonies were formed and observed, and the culture was terminated and the supernatant was discarded, followed by immersing twice with PBS. 4% paraformaldehyde was added to fix cells for 15 minutes. Then, fixing solution was removed, and the cells were stained with proper amount of Giemsa solution for 10-30 minutes. Then, cells were slowly washed off in dyeing solution with running water and air dried. The number of cells was calculated under microscopy.

### 2.9. Cell Migration and Invasion Assay

Matrigel (1 mg/ml, Solarbio) was diluted and prepared in 100 *μ*l solution. The transwell had two chambers including the upper and the lower. The upper was placed at 37°C for 4-5 hours; this would induce Matrigel to form gel. 2 × 10^5^ U251 and U87 cells in 200 *μ*l serum-free medium were plated into the 6.5 mm transwell chamber, and the complete medium was added into the lower chamber. Cells were then incubated at 37°C for 24 hours. Methanol was applied to fix cells for 15 min, and then, 0.1% crystal violet was employed to stain cells for 40 min. The cell migration from the upper chamber to the lower chamber is induced due to the permeability of the membrane and the composition of the medium in the upper and lower chambers. The superfluous, i.e., nonmigrated, cells in the upper layer are erased, while the cells in the lower layer, i.e., cells that have migrated, are stained with crystal violet. The migration rate was calculated. Invading cells will invade the extracellular matrix, which in turn determines cell invasion. Cell numbers were counted under a light microscope.

### 2.10. Statistical Analysis

All data were presented as the mean ± standard deviation (SD) for experiments performed in triplicate. Unpaired or paired Student's *t*-test or one-way analyses of variance (ANOVA) were utilized to analyze results followed by Tukey's test to compare each group. Data were analyzed, and graphs were plotted by using GraphPad Prism version 6.0 software (GraphPad Software, Inc., La Jolla, CA, USA). *p* < 0.05 indicated a significant difference.

## 3. Results

### 3.1. MTDH and MYBL2 Are Overexpressed in Glioma Cells

**Figure 1:**
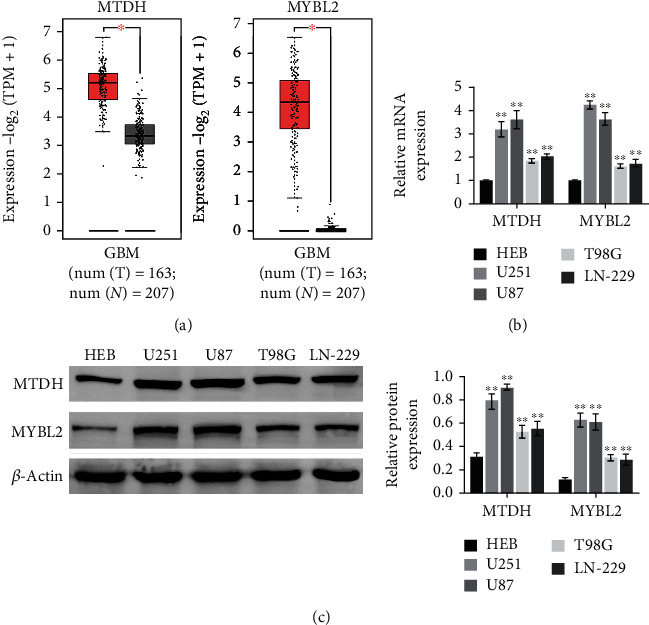
Differential expression analysis of MTDH and MYBL2 in normal tissues and glioma cancer tissues (a). Differential expression of two genes on the GEPIA online platform based on TCGA, the ordinate is the negative logarithmic transformation value of TPM+1 (b). In HEB, U251, U87, T98G, and LN-229 five cell lines. The relative expression levels of two genes in HEB, U251, U87, T98G, and LN-229 cell lines. *N* = 3, ^∗∗^*p* < 0.01 vs. the HEB group.

The predicted results are shown in Figure 1(a), indicating the significantly increased expression of MTDH and MYBL2 associated with different disease states (tumor or normal). To verify this result, qRT-PCR and WB were applied. The results showed that the expressions of these two genes in U251, U87, T98G, and LN-229 were significantly higher than those in HEB cells (Figure 1(b)). At the same time, the protein expression of MTDH and MYBL2 also showed consistent trend (Figure 1(c)). Notably, the expression levels of MTDH and MYBL2 were higher in U251 and U87, so they were used in subsequent experiments.

### 3.2. Knockdown of MTDH Inhibits MYBL2 Expression via FoxM1

**Figure 2:**
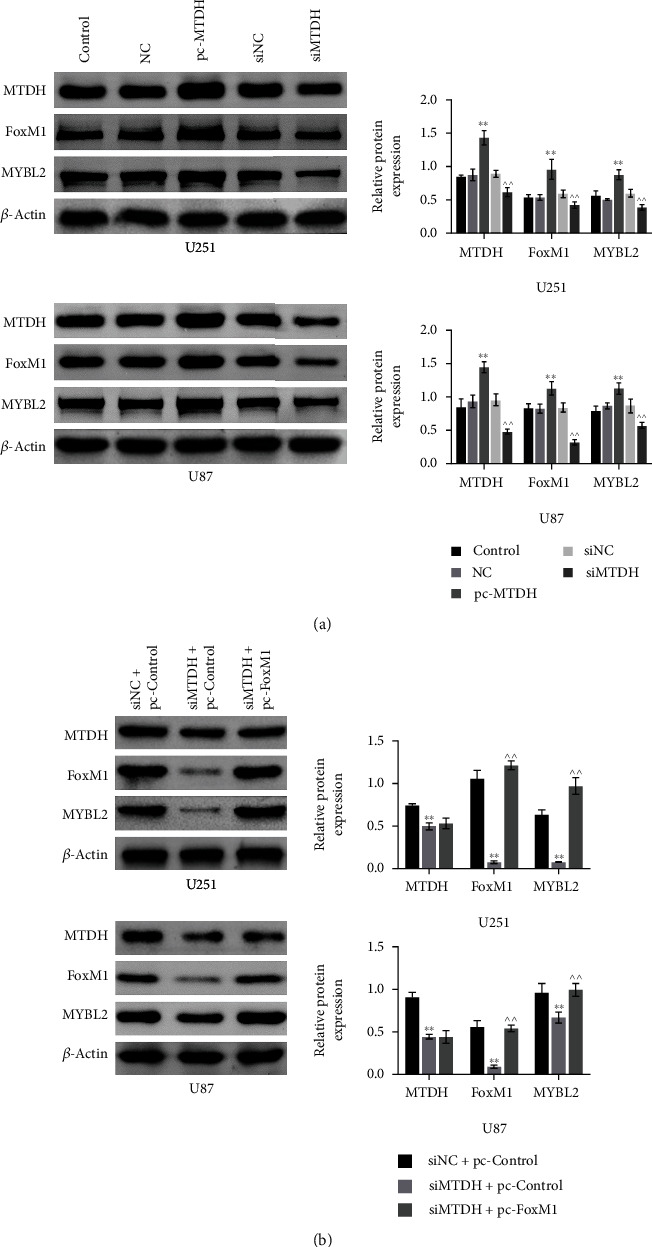
The protein expression of MTDH, MYBL2, and FoxM1 in each group in U251 and U87 cell lines (a). The protein bands of the three genes in the five groups of control, NC, pc-MTDH, and siMTDH and the histogram of the relative protein expression; *N* = 3, ^∗∗^*p* < 0.01 vs. the NC group; ^^^^*p* < 0.01 vs. the siNC group (b). The three genes in siNC+pc-control, siMTDH+. The histogram of protein bands and relative protein expression in the three groups of pc-control and siMTDH+pc-FoxM1. *N* = 3, ^∗∗^*p* < 0.01 vs. the siNC+pc-control group; ^^^^*p* < 0.01 vs. the siMTDH+pc-control group.

To investigate the role of MTDH in glioma cells, the expression of MTDH in U251 and U87 cells was knocked down. WB results showed that (Figure 2), in both cell lines, there was no significant difference in the expression of MTDH among the NC group, the siNC group, and the control group. The expressions of MTDH, FoxM1, and MYBL2 in the siMTDH group were significantly lower than those in the siNC group while the expression of MTDH, FoxM1, and MYBL2 in the pc-MTDH group was higher than that in the NC group (Figure 2(a)). These indicated the interplay among MTDH, FoxM1, and MYBL2. To investigate the gene targeted by MTDH, the protein expression of FOXM1 and MYBL2 in these two cell lines was subsequently examined. Through the knockdown of MTDH, it was found that the expression of MTDH, FoxM1, and MYBL2 in the siMTDH+pc-control group was significantly lower than that in the siNC+pc-control group but elevated expression of FOXM1 synergized with increased expression of MYBL2 was observed in the siMTDH+pc-FoxM1 group (Figure 2(b)).

### 3.3. Knockdown of MTDH Inhibits Glioma Cell Proliferation by Downregulating MYBL2

**Figure 3:**
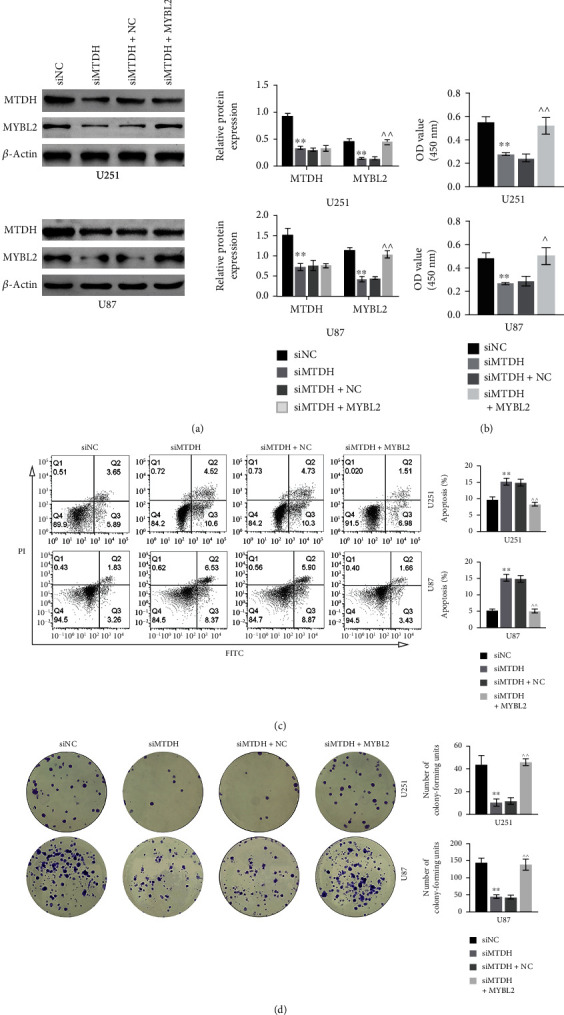
In U251 and U87 cell lines, the protein expression of MTDH and MYBL2 in four groups of siNC, siMTDH, siMTDH+NC, and siMTDH+MYBL2 and the proliferation, apoptosis, and clone formation of the four groups (a). Histograms of protein expression bands and relative protein expression of two genes in four groups (b). Cell proliferation in four groups (c). Cell grouping and calculated cells in four groups. Statistical graph of apoptosis rate. (d) Under the microscope, the colony formation situation of the four groups and the statistical graph of the calculated colony forming unit. *N* = 3, ^∗∗^*p* < 0.01 vs. the siNC group; ^^^^*p* < 0.01 vs. the siMTDH+NC group.

The effect of knockdown of MTDH on the proliferation of glioma cells was then examined. As shown in Figure 3(a), WB results showed that the expressions of MTDH and MYBL2 in siMTDH were significantly lower in the siMTDH group than those in the siNC group. At the same time, compared to the siMTDH+NC group, the addition of MYBL2 increased the expression of MTDH. This result was further verified using CCK-8 (Figure 3(b)); that is, in these two cell lines, the cell proliferation rate of siMTDH was significantly lower than that in the siNC group, and the proliferation rate was higher in the siMTDH+MYBL2 group than that in the siMTDH+NC group. In addition, the results of flow cytometry showed that the apoptosis rate of the siMTDH group was relatively high, which indicated that the knockdown of MTDH increased cell apoptosis while the addition of MYBL2 inhibited cell apoptosis compared to the siMTDH+NC group (Figure 3(c)). Colony formation assay showed that compared to the siNC group, decreased expression of MTDH reduced the colony formation in the siMTDH group, whereas the addition of MYBL2 reversed this effect (Figure 3(d)).

### 3.4. Knockdown of MTDH Inhibits Glioma Cell Migration and Invasion by Downregulating MYBL2

**Figure 4:**
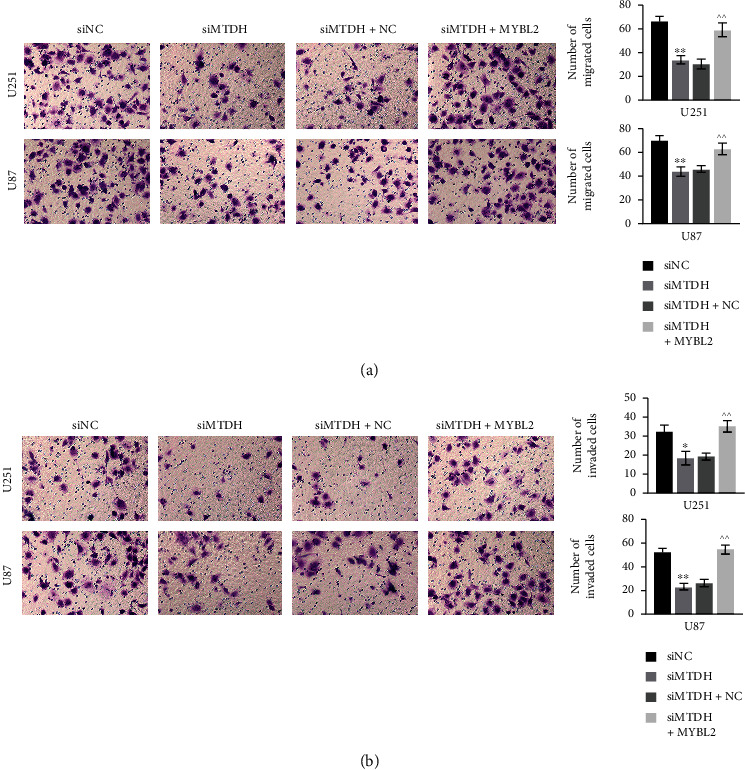
Cell migration and invasion in four groups, siNC, siMTDH, siMTDH+NC, and siMTDH+MYBL2, observed under the microscope in U251 and U87 cell lines (a). The cell migration in the four groups was observed under the microscope and the statistics of the calculated number of migrated cells (b). The cell invasion in the four groups was observed under the microscope and the calculated number of invasive cells. *N* = 3, ^∗^*p* < 0.05 vs. the siNC group; ^∗∗^*p* < 0.01 vs. the siNC group; ^^^^*p* < 0.01 vs. the siMTDH+NC group.

The cell migration and invasion of glioma cells U251 and U87 were detected after knockdown of MTDH. The results showed that the knockdown of MTDH decreased the cell migration compared to the siNC group, but in the siMTDH+MYBL2 group, the addition of MYBL2 enhanced cell migration compared to the siMTDH+NC group (Figure 4(a)). The cell invasion in each group showed the similar trend (Figure 4(b)).

## 4. Discussion

Glioma is a common primary tumor in the brain. Although many cancer therapies have been developed in the past few decades, the unique structure of the central nervous system in the brain has brought challenges to clinical treatment. Targeted therapy presents certain opportunities for cancer treatment [20]. In this study, we explored the function of MTDH in glioma cells and found that MTDH and MYBL2 were highly expressed in glioma tissues based on the analysis of the GEPIA website. To further validate this result, both qPCR and WB results confirmed that these two genes were expressed at higher levels in glioma cell lines. Subsequently, in order to study the role of MTDH in glioma, it was overexpressed and knocked down. WB results showed that both overexpression and knockdown had high efficiency, and the expression of target genes FoxM1 and MYBL2 was detected. The results showed that the expression of MTDH affected the expression of these two genes. In order to investigate whether the knockdown of MTDH would affect the proliferation and apoptosis of glioma cells, WB, CCK-8, and flow cytometry were subsequently performed. Our results showed that knockdown of MTDH decreased the proliferation rate of glioma cells and increased the rate of apoptosis, while MYBL2 had the opposite effect, indicating that knockdown of MTDH could inhibit the proliferation and increase the apoptosis of glioma cells by downregulating MYBL2. Finally, cell migration and invasion assay results showed that knockdown of MTDH reduced the ability of glioma cells to invade and migrate, while MYBL2 was able to reverse this inhibition, indicating that knockdown of MTDH inhibited glioma cell migration and migration by downregulating MYBL2.

MYBL2 is a member of the myeloblastosis transcription factor family that is activated in tumorigenesis and progression as Rachel's systemic review summarised [21]. In breast cancer, the increased expression of MYBL2 regulates the changes in downstream microRNAs and single nucleotides in the coding regions of other genes. Relevant studies have shown that MYBL2 is a key downstream factor of the Akt/FoxM1 signaling pathway in promoting human glioma progression and can be used as a new candidate gene for glioma molecular targeted therapy and a biomarker for radiotherapy [18]. This was verified in the present study, where knockdown of MTDH downregulates MYBL2, suggesting that it may play a role in the treatment of glioma. The role of MTDH in this study has also been validated in multiple studies. For example, Zhang et al. revealed that MiR-30b-5p regulates glioma cell proliferation by directly targeting MTDH [22]. Tong et al. showed that overexpressed glioma cell-associated activation gene promotes a series of signaling pathways that promote gliomas [12]. Therefore, knocking down MTDH and targeting MYBL2 may provide new insights for glioma therapy.

There are limitations to this study. MTDH has been shown to be aberrantly expressed in glioma and has been shown to regulate EMT during cancer invasion. The mechanism involved in this process may be through regulation of certain miRNAs and activation of Akt or coactivation of key signaling pathways with target genes. MYBL2 has also been proved to be associated with poor prognosis of cancer by pan-cancer analysis, and some literatures have shown that MYBL2 can be upregulated by activation of Akt/FoxM1 to promote the development of glioma. However, it is still unclear through which biological microevents/processes MTDH affects MYBL2, thus affecting the behavior of cells. These will be explored in the future studies. It is still not clear whether MTDH acts directly on MYBL2 or through other proteins that mediate it. But it is worth affirming that the decreased expression of MTDH will mediate the downregulation of MYBL2. Moreover, this study has not established an animal glioma model, and a good animal model will provide some evidence for targeting MTDH in the treatment of glioma, which will be conducted in the following studies.

## 5. Conclusion

This study showed that MTDH and MYBL2 were overexpressed in gliomas, and knockdown of MTDH inhibited the expression of MYBL2. Further experiments showed that knockdown of MTDH inhibited the proliferation, migration, and invasion of glioma cells and promoted cell apoptosis. Overexpression of MYBL2 could reverse the knockdown effects of MTDH in glioma cells. These results suggested that downregulation of MTDH inhibited the progression of glioma cancer.

## Figures and Tables

**Table 1:** 

Gene	Sense	Antisense
*MTDH*	GACCTAGCCCAGCTGAAGAAT	TTTGCAGTTATACTTCGGGGA
*FoxM1*	ATACGTGGATTGAGGACCACT	TCCAATGTCAAGTAGCGGTTG
*MYBL2*	CTTGAGCGAGTCCAAAGACTG	AGTTGGTCAGAAGACTTCCCT
*β-Actin*	CATGTACGTTGCTATCCAGGC	CTCCTTAATGTCACGCACGAT

## Data Availability

All data generated or analyzed during this study are included in this published article.

## Abbreviations

ANOVA: Analyses of variance
BCA: Bicinchoninic acid
CCK-8: Cell counting kit 8
DMEM: Dulbecco's modified Eagle medium
EMT: Epithelial–mesenchymal transition
FITC: Fluorescein isothiocyanate
FoxM1: Forkhead box M1
GEPIA: Gene expression profiling interactive analysis
HRP: Horseradish peroxidase
MTDH: Metadherin/astrocyte-elevated gene-1
MYBL2: MYB protooncogene-like 2
PI: Propidium iodide
PVDF: Polyvinylidene fluoride or polyvinylidene difluoride
qRT-PCR: Real-time quantitative reverse transcription PCR
SD: Standard deviation
SDS-PAGE: Sodium dodecyl sulfate–polyacrylamide gel electrophoresis
TCGA: The Cancer Genome Atlas
TME: Tumor environment
WB: Western blot.
